# Rush Medical College Clinic

**Published:** 1857-12

**Authors:** 


### RUSH MEDICAL COLLEGE CLINIC.

The Clinic held at the College every Wednesday and Saturday morning by Professors Brainard and Byford, has been well attended during the present College term; and the number of highly-interesting surgical cases, requiring important operations, have been unusually large. Three of these cases are reported in the present number of the *Journal*, and our readers may expect a series of reports from the same source.

